# Nosocomial Buffalopoxvirus Infection, Karachi, Pakistan

**DOI:** 10.3201/eid1306.061068

**Published:** 2007-06

**Authors:** Afia Zafar, Robert Swanepoel, Roger Hewson, Mazhar Nizam, Altaf Ahmed, Akhtar Husain, Antoinette Grobbelaar, Kevin Bewley, Valerie Mioulet, Barry Dowsett, Linda Easterbrook, Rumina Hasan

**Affiliations:** * Aga Khan University Hospital, Karachi, Pakistan; † National Institute for Communicable Diseases, Sandringham, South Africa; ‡ Centre for Emergency Preparedness and Response, Porton Down, Salisbury, UK; § Patel Hospital, Karachi, Pakistan

**Keywords:** Buffalopoxvirus, nosocomial outbreak, multicenter, burn patients, dispatch

## Abstract

During 5 months in 2004–2005, buffalopoxvirus infection, confirmed by virus isolation and limited nucleic acid sequencing, spread between 5 burns units in Karachi, Pakistan. The outbreak was related to movement of patients between units. Control measures reduced transmission, but sporadic cases continued due to the admission of new patients with community-acquired infections.

Buffalopoxvirus, a strain of *Vaccinia virus* in the genus *Orthopoxvirus* of the family *Poxviridae,* has been associated with sporadic cases and outbreaks of infection in Asian buffalo (*Bubalus bubalis*) in Pakistan, India, Bangladesh, Russia, Indonesia, Egypt, and Italy (*1*–*13*). The virus causes pock lesions on the udder, which adversely affect milk production and can be a source for human infection characterized by transient fever, regional lymphadenitis, and pock lesions, usually on the hands, from contact with infected buffalo.

## The Study

Karachi is the largest city in Pakistan, with a population of 12 million. Healthcare is provided by public and private hospitals, and there are 5 major burns units. In January 2005, pustular lesions were observed on the foot of a patient in 1 of the burns units, and similar lesions subsequently appeared on other patients. Local health authorities were informed of the outbreak, and an investigatory team confirmed reports of similar infections in the city’s other burns units, with retrospective identification of at least 19 probable cases occurring over a 5-month period.

**Figure 1:**
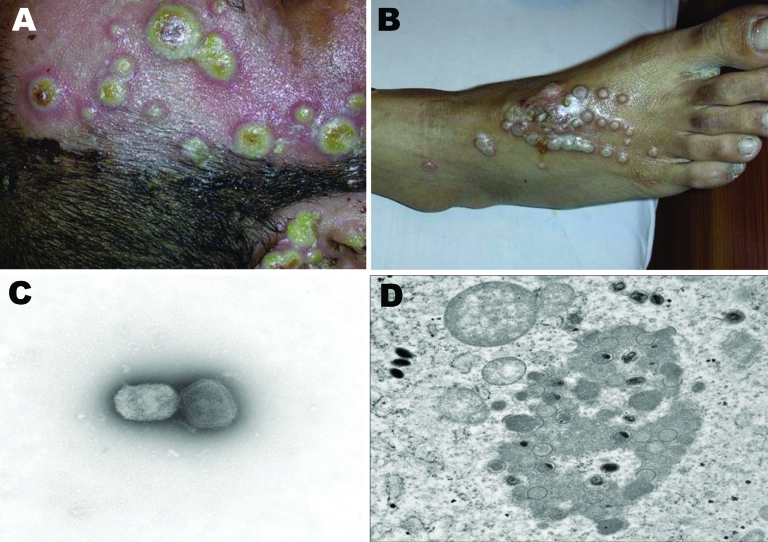
Nosocomial buffalopoxvirus infection of patients in burns units. A) Lesions involving intact skin around a burn wound and the wound itself. B) Lesions around an insertion site for an intravenous line. C) Orthopoxvirus particles detected by electron microscopy (EM) examination of negatively stained grids prepared from pustular material (magnification ×73,000). D) Transmission EM examination of ultrathin sections of infected Vero cell cultures showing classic intracytoplasmic orthopoxvirus factories and maturing virus particles (magnification ×21,000).

Most patients had a fever (39.0°C–40.5°C) for 2–3 days, followed by the appearance of an eruption(s). Lesions were typically small, rounded, umbilicated, and nodular in appearance with an erythematous base (Figure 1A). They contained cheesy pustular material and increased in size (from 1–2 mm to >1 cm in diameter) and severity over 7–8 days. The lesions involved burn wounds and intact skin surrounding them, with the unhealed margins of the wounds being covered by a layer of thick yellow secretion. Some patients had a sparse rash; others had closely spaced lesions that produced a cobblestone appearance. The lesions developed crusts, which shrank and sloughed without residual scars. A single lesion developed on a paramedical staff member’s finger, and lesions developed around 1 patient’s insertion site for an intravenous line (Figure 1B). In all instances, the disease was self limiting, and patients recovered in 3–4 weeks.

Results of bacteriologic and mycologic examination of biopsy samples, impression smears, and swab samples from lesions were negative. Histopathologic examination showed extensive ulceration and granulation, with epidermal necrosis and subepidermal edema plus acute and chronic inflammatory cell infiltration. No molluscum bodies were observed, but eosinophilic cytoplasmic inclusions were present in keratinocytes. Impression smears and biopsy tissues were sent to the Special Pathogens Unit, National Institute for Communicable Diseases (NICD), Sandringham, South Africa, and to the Health Protection Agency (HPA), Centre for Emergency Preparedness and Response, Porton Down, Salisbury, United Kingdom. Electron microscopy of negative-stained grids prepared at HPA and NCID laboratories from pustular material showed orthopoxvirus particles, and examination of ultrathin sections prepared from infected Vero cell cultures at HPA found classic orthopox intracytoplasmic virus factories and particle maturation sites (Figure 1C, D).

**Figure 2:**
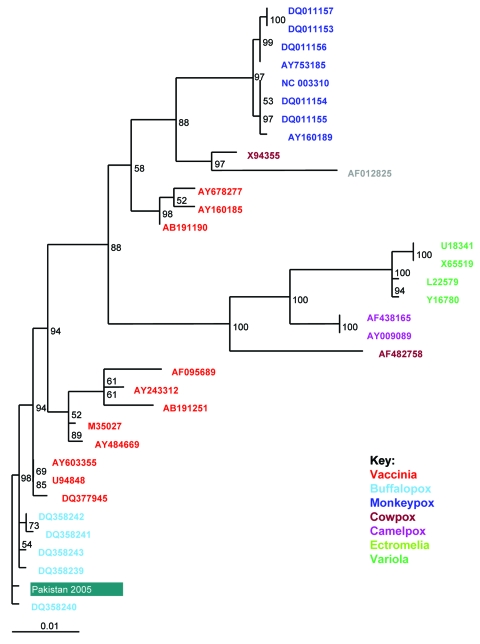
Maximum likelihood phylogenetic tree based on a 955-nt alignment of the Karachi isolate and 33 orthopoxvirus sequences of the B5R gene from GenBank constructed with ClustalW (www.ebi.ac.uk/clustalw/index.html) and TREE-PUZZLE (http://bioweb.pasteur.fr/seqanal/interfaces/puzzle.html); figures at nodes represent PUZZLE support values. The orthopoxvirus types are indicated to the right. The Karachi isolate sequence (Pakistan 2005) groups within the buffalopox B5R genes.

PCR was performed on nucleic acid extracted from the samples, using primers specific for regions of the orthopoxvirus hemagglutinin gene (at NICD) and B5R membrane protein gene (at HPA). After nucleotide sequences were determined for the PCR products, phylogenetic analyses were conducted in relation to corresponding orthopoxvirus sequences obtained from GenBank, using methods described elsewhere (*14*,*15*). The causative agent was found to cluster with buffalopoxviru*s* isolates within the vaccinia subgroup of orthopoxviruses (Figure 2), and 3 patients from 2 separate burns units were shown to be infected with the identical virus, which was distinct from other known buffalopoxvirus isolates. To investigate the possibility of a shared source of infection, 17 samples of saline, antimicrobial drug ointments, petroleum jelly, cotton dressings, and swabs in common use were obtained from the 5 burns units and tested by PCR at NICD; no results were positive. Inquiries led to the suggestion that the outbreak was probably propagated by transfer of infected patients between burns units. This hypothesis was confirmed when a policy to isolate all new admissions, including referrals from other burn centers, for their first 2 weeks in a unit successfully controlled transmission of new endogenous cases.

Control measures included education of staff, single-room or cohort isolation of patients infected or suspected of being infected with buffalopoxvirus, and reinforcement of infection-control practices, such as hand disinfection after contact with any patient. To reduce virus load in the environment, the facilities were cleaned more frequently and hypochlorite disinfectant was used for cleaning. The measures proved effective in reducing transmission within burns units, but they did not prevent the sporadic arrival of newly infected patients.

## Conclusions

Buffalopoxvirus outbreaks reported to date have been geographically restricted, and human cases have been limited to persons with direct exposure to infected animals, usually in rural communities (*1*–*11*). This reported outbreak uniquely involved nosocomial infections in 5 widely separated burns units in Karachi, Pakistan. However, buffaloes are the most common dairy animal in Pakistan, even within the city limits of Karachi, and buffalo fat, particularly in the form of butter or ghee, sometimes is used at home as a dressing for burns. Thus, burn patients newly infected with buffalopoxvirus may periodically arrive at burns units. Due to disparity in the sophistication and cost of the care provided at the burns units in Karachi, patients are often transferred or move themselves between units, thus facilitating the possible spread of infection. In this outbreak, 6 of the 19 patients with putative cases of buffalopoxvirus infection are known to have transferred between burns units during treatment. Fortunately, the infection was of low virulence for humans.

Delay in recognizing and investigating the outbreak is cause for concern and can be ascribed to poor awareness and lack of resources. Clearly, improvements are needed in disease surveillance, diagnostics, and infection control.
